# Nitric oxide therapy for dermatologic disease

**DOI:** 10.4155/fso.15.37

**Published:** 2015-08-01

**Authors:** Brandon L Adler, Adam J Friedman

**Affiliations:** 1 Department of Medicine (Division of Dermatology), Albert Einstein College of Medicine, Bronx, NY, USA; 2 Department of Physiology & Biophysics, Albert Einstein College of Medicine, Bronx, NY, USA; 3 George Washington School of Medicine & Health Sciences, Washington, DC, USA

**Keywords:** cosmeceutical, nitric oxide synthase, NOS, reactive nitrogen oxide species, RNOS, skin and soft-tissue infection, SSTI

## Abstract

Nitric oxide (NO) plays an important role in the maintenance and regulation of the skin and the integrity of its environment. Derangement of NO production is implicated in the etiology of a multitude of dermatologic diseases, indicating future therapeutic directions. In an era of increasing resistance rates to available antibiotics and subpar development of new agents, NO is promising as a prospective topical broad-spectrum antimicrobial agent with small likelihood of resistance development. Because the greatest strides have been made in the setting of infectious disease and skin and soft-tissue infection, this will be a major focus of this article. In addition, we will review NO's role in skin regulation and dysregulation, immune function, the various topical release systems that have been devised and tested, NO's relation to UV radiation and skin pigmentation, and finally, its potential applications as a cosmeceutical.

## Abstract (lay abstract)

Nitric oxide (NO) is molecule that plays many roles in both normal and abnormal skin processes. There is strong evidence that NO-releasing materials may serve as new, unique antimicrobial agents, at a juncture when current antibiotics are confounded by increasingly resistant microorganisms. We review the numerous functions NO serves in skin physiology, focusing on its antimicrobial capabilities, and present evidence for its potential utility as a cosmetic agent.

**Figure 1.**
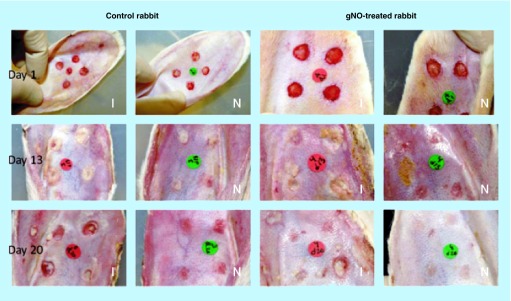
**Infected full-thickness dermal wounds on New Zealand white rabbit ears.** Ischemic (I) and nonischemic (N) wounds were treated with NO probiotic patch or control patch at days 1, 13, and 20 postsurgery. gNO: Gaseous nitric oxide. Reproduced with permission from [39]. © John Wiley & Sons, Inc. (2012).

**Figure 2.**
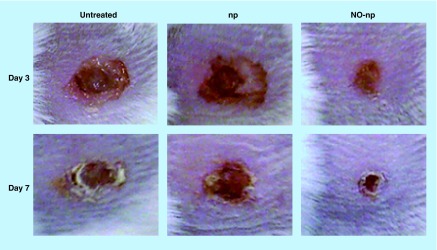
**Methicillin-resistant *Staphylococcus aureus*-infected full-thickness wounds in mice at days 3 and 7: untreated, treated with empty nanoparticles and treated with nitric oxide nanoparticles.** NO: Nitric oxide; np: Nanoparticle. Reproduced with permission from [46]. © Macmillan Publishers Ltd. (2009).

**Figure 3.**
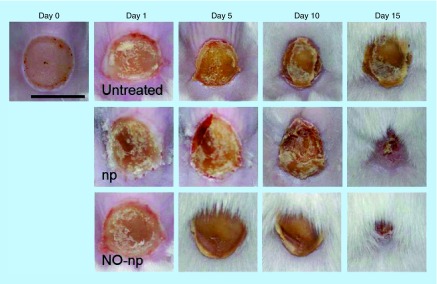
**Burn wounds infected with *Candida albicans*: untreated, treated with empty nanoparticles, and treated with nitric oxide nanoparticles, at days 0, 1, 5, 10 and 15.** Scale bar: 5 mm. NO: Nitric oxide; np: Nanoparticle. Reproduced with permission from [48]. © Macherla C, Sanchez DA, Ahmadi MS *et al*. (2012).

**Figure 4.**
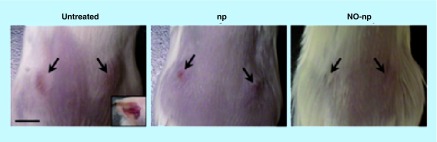
**Methicillin-resistant *Staphylococcus aureus*-infected murine abscesses: untreated, treated with empty nanoparticles, and treated with nitric oxide nanoparticles at day 4.** Arrows denote abscesses. Inset: representative purulent abscess 4 days post-methicillin-resistant *Staphylococcus aureus* infection. Scale bar: 5 mm. NO: Nitric oxide; np: Nanoparticle. Reproduced with permission from [49]. © Han *et al*. (2009).

**Figure 5.**
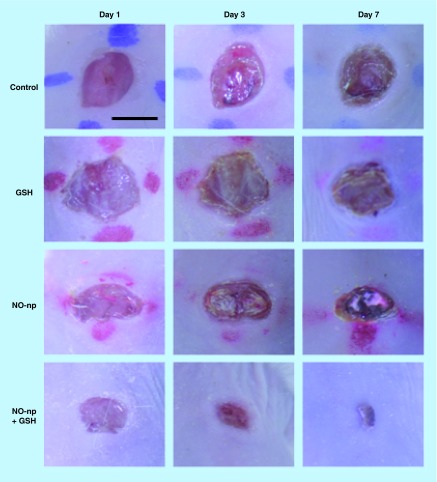
***Pseudomonas aeruginosa*-infected excisional wounds in mice: untreated, glutathione-treated, nitric oxide nanoparticle-treated and nitric oxide nanoparticle + glutathione-treated at days 1, 3 and 7.** Scale bar: 5 mm. GSH: Glutathione; NO: Nitric oxide; np: Nanoparticle. Reproduced with permission from [52]. © *J. Drugs Dermatol.* (2012).

**Figure 6.**
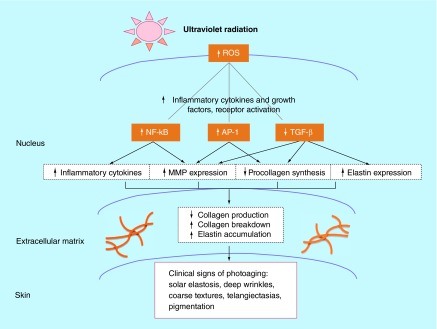
**Role of reactive oxygen species in photoaging.** MMP: Matrix metalloproteinase; ROS: Reactive oxygen species. Reprinted with permission from [57]. © Elsevier (2004).

## Nitric oxide & skin

Nearly every member of the skin cell population expresses nitric oxide synthase (NOS) and is thereby able to produce NO to accomplish essential physiologic processes. This body of cells includes keratinocytes, endothelial cells, fibroblasts, melanocytes, adipocytes, Langerhans cells, neutrophils and macrophages. The broad distribution of skin-situated NO producers enables the molecule's participation in vital cutaneous physiologic processes, including formation of a protective barrier and antimicrobial defense, establishment and maintenance of circulation, and melanogenesis and erythema in response to ultraviolet light exposure [1–3].

The production of endogenous NO is handled by three isoforms of NOS. Two are constitutively expressed: NOS1/neuronal NOS (nNOS) and NOS3/endothelial NOS (eNOS), calcium-dependent enzymes that are regulated by calmodulin. Once activated, they produce small amounts of NO over brief periods of time. NOS2/inducible NOS (iNOS), on the other hand, generates large quantities of NO by a noncalcium-dependent mechanism. It is stimulated by bacterial products, cytokines and neuropeptides [1,2].

### Dichotomous role of NO in dermatologic physiology & pathology

NO is a Janus-faced molecule, playing beneficial as well as harmful roles in routine skin physiology and states of dysregulation and disease. Given NO's crucial role in the basic integrity and day-to-day functioning of the skin, it is unsurprising that derangements in production, signaling, or both are associated with disease states. A fine balance must exist in NOS expression and subsequent NO production, as both excess and dearth are linked to pathology. Additionally, as discussed below, endogenous NO production has antimicrobial functions and may be augmented by topical release systems, with attendant consequences for future management of skin and soft-tissue infection.

#### An overview of NO & dermatologic disease

Inappropriate iNOS upregulation is implicated in such wide-ranging pathophysiologic conditions as:

Cutaneous lupus erythematosus [3];
Inflammatory skin disorders: psoriasis, atopic dermatitis, irritant and allergic contact dermatitis [4–6];
Keloids [7];
Morphea/scleroderma [8];
Pemphigus vulgaris [9];
Pityriasis lichenoides [10];
Sjögren's syndrome [11];
Stevens-Johnson syndrome/toxic epidermal necrolysis [12].

Regulation of iNOS expression may occur at various levels, including promoter activity, mRNA and protein stability, enzyme activity and substrate supply. Glucocorticoids and TGF-β downregulate iNOS, and it is thought that a negative feedback loop exists through which NO itself inhibits iNOS expression and activity. Nevertheless, the lack of clinically effective self-regulation of NO in these dysfunctional conditions is a pertinent topic for further inquiry. It should stimulate the search for new therapeutic targets to treat many of these chronic and debilitating conditions. For instance, the NOS inhibitor L-NMMA in aqueous cream BP was applied to psoriatic lesions and showed 77% inhibition of NO production as well as inhibition of angiogenesis as compared with control. However, the therapeutic gain of this approach was minimal, with insignificant differences in erythema and skin thickness as compared with vehicle [1,9,13–16].

In contrast to the conditions listed above that are characterized by NO overproduction, states of excessive vascular tone – although multifactorial – may relate to NO deficiency [17]. NO was originally termed endothelium-derived relaxing factor (EDRF), owing to its vasodilatory function. Therefore, NO donors have a place in the treatment of vasospastic disease, notably Raynaud phenomenon. This condition is characterized by exaggerated digital vascular spasm in response to cold exposure or strong emotion, leading to color changes in the overlying skin and ischemic symptoms that range from achiness and paresthesias to severe pain and ulceration. A recent multicenter, double-blind, randomized, placebo-controlled crossover study examined a novel topical nitroglycerine gel preparation in Raynaud patients, and found significant improvement in skin blood flow compared with placebo [18].

As experimental data accumulates and our knowledge base continues to grow, it is becoming clearer how disruptions of NO production on even a micro scale can lead to overt dermatologic disease. Although still in their infancy, targeted therapies could act to restore NO homeostasis, thereby alleviating the symptoms of these oftentimes debilitating conditions. At present, much more experimental evidence is available in the infectious disease/microbiology arena, in which the same idea of a necessary fine balance of NO production persists – with even more promising future therapeutic applications.

#### NO as an antimicrobial agent

NO exerts antimicrobial activity in a concentration-dependent bimodal fashion. At low concentrations it stimulates the immune system by enhancing immune cell proliferation, differentiation, and apoptosis, as well as cytokine production, expression of adhesion and co-stimulatory factors, and extracellular matrix constituent synthesis and deposition.

Conversely, iNOS, as a component of the innate immune system, produces large quantities of NO when activated by bacterial polysaccharides and endotoxins as well as proinflammatory cytokines. At concentrations greater than 1 μM, NO's utility in combating pathogens derives largely from its ability to react with reactive oxygen intermediates to generate reactive nitrogen oxide species (RNOS). Peroxynitrite (OONO^-^), the most reactive and cytotoxic RNOS, forms when NO interacts with superoxide. RNOS possess numerous antimicrobial properties, including:

Induction of nitrosative and oxidative stress;
Inactivation of essential enzymes;
Depletion of intracellular iron stores;
Damage to microbial DNA, directly and indirectly (via generation of alkylating agents and hydrogen peroxide and inhibition of DNA repair);
Disruption of the microbial cell membrane through lipid peroxidation [2,19–20].

iNOS knockout mice demonstrate increased susceptibility to infection with herpes simplex virus as well as reduced clearance of latent virus [21]. When mice were treated with an iNOS inhibitor, they proved more vulnerable to infection with intracellular bacteria [22].

Perhaps the most significant aspect of NO's microbicidal activity is the lack of demonstrated resistance to date. This is thought to relate to NO's multiple mechanisms of action, to overcome which would require the simultaneous development of distinct resistance mutations. Some minor compensatory mechanisms against the actions of NO have been elucidated; however, no protection against the high concentrations of NO released by donating materials is evident. Isolates of *Staphylococcus aureus*, MRSA, *Staphylococcus epidermidis, Escherichia coli* O157:H7 and *Pseudomonas aeruginosa* that survived exposure to lethal NO concentrations showed no increase in minimum inhibitory concentration levels afterward [23].

## Topical release systems

NO represents a double-edged sword: although both over- and under-production are implicated in numerous diseases, appropriately harnessing its antimicrobial power for treatment purposes would be valuable in view of rising antibiotic resistance rates. Due to its lipophilicity, NO easily traverses the stratum corneum, the outer layer of the epidermis. To be exploited therapeutically, however, it must be integrated into a stable delivery platform capable of reliable cutaneous release of predictable and microbicidal quantities of NO over a sustained time period and with minimal side effects. Meeting these criteria would enable full realization of NO's potential to serve as a broad-spectrum topical antimicrobial agent. Although these systems are discussed at length in other articles in this issue, Table 1 lists the major classes of agents tested for antimicrobial capability along with their strengths and weaknesses and the organisms against which they have shown activity. Taken in sum, the various NO-releasing technologies demonstrate NO's activity against an impressive array of organisms, including Gram-positive and -negative bacteria, fungi and parasites [2,19,24].

## NO for the treatment of skin & soft-tissue infection

Skin and soft-tissue infection (SSTI) due to community-acquired methicillin-resistant *S. aureus* increased drastically in the period between 1995 and 2005. *S. aureus* is isolated in >80% of cultured pathogen-positive SSTIs; MRSA represents around half of those isolates. The incidence of SSTI caused by Gram-negative pathogens is substantial, found in two large population-based studies to compose 14 and 28% of isolates, respectively. However, these figures must be interpreted warily in light of low SSTI culture rates: in both studies, only 23% of cases had an accompanying specimen [25,26].

An acronym was developed for a dangerous group of resistant organisms able to ‘escape’ many (or most) members of our antibiotic armamentarium: the ESKAPE pathogens (*Enterococcus faecium, S. aureus, Klebsiella pneumoniae, Acinetobacter baumannii, Pseudomonas aeruginosa, Enterobacter* spp.). Antibiotic use continues to increase unchecked, extending in some cases to agents of last resort (e.g., polymyxins). In spite of skyrocketing resistance rates, development of new antibiotics grows more stagnant by the year. There is dire need for novel nonantibiotic antimicrobial agents to stem the rising tide of resistant infection. As seen in Table 1, many resistant bacteria are susceptible to the effects of NO [27].

NO-releasing materials represent a source of hope in the losing war on resistant SSTI-causing organisms:

Acidified nitrite creams have demonstrated activity *in vitro* against *B. cepacia, P. aeruginosa, S. aureus* and *C. albicans* [28,29].
Among the diazeniumdiolates (NONOates), β-Gal-NONOate exhibited greater bactericidal activity against *E. coli* than conventional NONOates [30]. In addition, DETA-NO inhibited multiple *Candida* species’ growth, and proves synergistic in combination with azole antifungals [31]. Schoenfisch *et al*.'s NONOate-covered silica nanoparticles have proven *in vitro* efficacy against *P. aeruginosa* [32]. They also showed strong antibiofilm activity against *E. coli, P. aeruginosa, S. aureus, S. epidermidis* and *C. albicans* [33].
Ghaffari *et al*.'s gaseous NO (gNO) chamber was bactericidal against *P. aeruginosa* and *S. aureus* with constant exposure at 160 ppm [34]. Twenty-four hours of constant gNO exposure at 200 ppm was bactericidal against *E. coli, P. aeruginosa, S. aureus*, MRSA, Group B *Streptococcus* and *C. albicans* [35]. Intermittent gNO exposure at 160 ppm also demonstrated bactericidal effects against *E. coli, P. aeruginosa and S. aureus*, albeit requiring 10 more hours of exposure for the desired antimicrobial outcome [36]. *In vivo*, full-thickness rabbit wounds inoculated with *S. aureus* treated with gNO at 200 ppm for 8 h daily over 3 consecutive days led to significant decrease in bacterial burden [37].
The NO probiotic patch was bactericidal against *A. baumannii, E. coli, P. aeruginosa, S. aureus* and MRSA, exhibiting near-complete eradication of the latter *in vitro* [38]. In full-thickness rabbit wounds infected with *S. aureus*, probiotic patch application resulted in significantly decreased wound area but no significant attenuation of bacterial burden versus controls (Figure 1) [39].
Two *S-*nitrosothiols (RSNOs) – GSNO (*S-*nitrosoglutathione) and SNAC (*S-*nitroso-*N-*acetylcysteine) demonstrated effective bacterio-static and -cidal activity against *Enterobacter aerogenes, P. aeruginosa, Serratia marcescens, S. aureus* and coagulase-negative staphylococci. In all organisms evaluated, the antimicrobial activity of SNAC exceeded that of GSNO [40]. Both agents also exhibit activity against *Leishmania major* and *L. amazonensis* [41]. Another RSNO, SNAP (*S-*nitroso-*N*-penicillamine), led to visible improvement within 5 days in 11 patients with cutaneous leishmaniasis. All treated lesions were healed by 30 days after treatment initiation, compared with no improvement in vehicle-treated patients [42].
NO-releasing zeolites have demonstrated activity against *C. difficile, P. aeruginosa, S. aureus* and MRSA [43], as well as *Bacillis subtilis* and *E. coli* [44].
Hybrid hydrogel/glass composite NO-releasing nanoparticles (NO-np) were efficacious *in vitro* against a variety of Gram-positive and -negative organisms, including *A. baumannii, Enterococcus faecalis, E. coli, K. pneumoniae, S. aureus*, MRSA and *Streptococcus pyogenes*. They also demonstrated *in vitro* activity against *C. albicans* [45–48].

To date, the most extensive preclinical *in vivo* work in this arena has been performed with the NO-np; therefore, these investigations will be reviewed. When NO-np were applied to a murine model of MRSA-infected full-thickness wounds, there was clinically evident acceleration of wound closure (Figure 2) and decreased bacterial concentration in the treatment arm as compared with controls. It was apparent histologically that NO-np-treated wounds exhibited decreased signs of inflammation and dermal destruction and more well-formed granulation tissue versus controls [46].

Similar results were obtained in a murine model of multidrug-resistant *A. baumannii*-infected full-thickness excisional wounds. The rate of wound healing was accelerated as compared with the MRSA-infected wounds, and inhibition of collagen degradation was especially prominent in the wounds treated with NO-np. *A. baumannii*, traditionally associated with infection following war trauma in Iraq and Afghanistan, is growing in prominence domestically in the setting of both nosocomial and community-acquired infection; it possesses the frightening ability to rapidly acquire antibiotic resistance [47].

In burn wounds infected with *C. albicans*, those treated with NO-np healed more rapidly than control (Figure 3), with signficantly lower fungal burden. On histological examination, there was less inflammation, more fibrin deposition, and increased collagen content in the NO-np-treated wounds [48].

Abscesses are notorious for their resilience in the face of most traditional antibiotics, owing to their poor perfusion and biofilm-like qualities. Both intradermal and intramuscular abscesses are commonly associated with MRSA infection; murine models of both were treated with hybrid NO-np. Both topical and intradermal application of NO-np significantly reduced intradermal abscess area (Figure 4) and bacterial burden. Histologically there was reduced inflammation and less destruction of dermal and subcutaneous structures [49]. In the intramuscular abscess model, NO-np significantly decreased MRSA load compared with both control animals and a vancomycin-treated group. There was clinically evident abscess resolution, assessed visually by decreased size and purulence compared with control. Histological examination of abscesses treated intralesionally with NO-np demonstrated decreased muscle necrosis and granulomatous inflammation as compared with control. Notably, the effect of vancomycin – commonly used in treatment of MRSA SSTI – on intramuscular abscesses was intermediate between untreated and NO-np groups [50].

Friedman *et al*. sought to determine the ability of hybrid NO-np to generate *S-*nitrosoglutathione (GSNO) in the presence of glutathione (GSH). GSNO is an *S-*nitrosothiol whose major action is nitrosation of sulfhydryl-containing cellular proteins, thereby reversibly blocking enzyme and protein functioning. Thus GSNO can be utilized for antimicrobial purposes, although bacteria are known to defend themselves via GSNO reductases and nitroreductases as well as GSH regeneration. In combination with GSH, the NO-np formed GSNO in significant concentrations over a time period greater than 24 h. This steady rate of GSNO formation is thought to result from the NO-np's controlled, sustained NO release over time. *In vitro*, NO-np + GSH exhibited significant bacterio-static and -cidal activity against *E. coli, K. pneumoniae* and *P. aeruginosa* compared with both control and solo NO-np. *K. pneumoniae* demonstrated the greatest resistance to the mixture, while *P. aeruginosa* was most susceptible, with no growth over 24 h [51].

In response to this finding, the combination platform was employed in the aforementioned murine excisional wound model, this time infected with multidrug-resistant *P. aeruginosa*. Treatment with NO-np + GSH resulted in significantly accelerated wound healing and lower bacterial burden clinically and histologically as compared with both control and NO-np-treated wounds (Figure 5). Both *in vitro* and *in vivo*, then, the antimicrobial activity of NO-np + GSH outweighed that of NO-np alone. The stability of GSNO as an NO reservoir as well as its strong nitrosating capability likely contributed to this finding. Furthermore, microbial systems used to import GSH actively take up GSNO as well, giving it access to bacterial targets inaccessible to free NO [52].

In aggregate, the above findings support the conclusion that NO-np represent a creative means of treating SSTI at a time in which antibiotic resistance rates are soaring. The successful translation of NO-np from bench to bedside would be a welcome achievement during the decline and fall of the antibiotic empire.

## NO & sun: attenuation of ultraviolet radiation-induced damage, UV-induced melanogenesis & possible cardioprotective effect

Pathogenic organisms are not the only entities hoping to breach the NO-fortified skin barrier. Ultraviolet radiation, a constant threat, induces keratinocytes to release NO in a sustained fashion. Both UVA and UVB stimulate this release, which aligns with the timing of UV-induced erythema, declining after around 3 days. This is to be expected, as UV-induced erythema is also related to NO, and can be prevented by intradermal injection of L-NAME, a selective inhibitor of NOS. It is thought that NO acts in this setting to attenuate the free radical damage that would result if UV-generated photoproducts had free reign. NO protects endothelial cells against the apoptotic effects of UVA by both enzymatic and non-enzymatic pathways. In the latter case, NO is generated from NO-related products contained in the epidermis, superficial dermis and sweat. In mice lacking eNOS, increased apoptosis is seen following ultraviolet radiation (UVR) exposure [1,13,53–54].

Skin pigmentation, especially subsequent to UV exposure, is also closely related to NO. As mentioned earlier, melanocytes express NOS. Following UVA and B exposure, keratinocytes co-cultured with melanocytes release NO, leading to cGMP upregulation and stimulation of tyrosinase, with melanin as end product (i.e., melanogenesis). This process can be inhibited by L-NAME. In another experiment, human melanocytes cultured in the presence of the NO donor *S*-nitroso-*N*-acetyl-L-arginine demonstrated increased tyrosinase mRNA expression as early as 2 h after administration, as well as a small but significant increase in tyrosinase protein level after 24 h and a nearly twofold increase in tyrosine hydroxylase activity after 4 consecutive days of exposure. Finally, such detailed features of pigmentation as melanocytes’ dendritic branching, eumelanin:pheomelanin ratio, and melatonin-induced aggregation of melanosomes, are all affected by NO. In a system so closely linked to one molecule, it follows that dysfunction of NO's regulatory effect may come into play during states of postinflammatory, endocrine, and hereditary hypo- and hyper-melanosis [1,13,53,55].

Sunlight may also lead to NO-induced effects that are more than skin deep. Following UVA exposure, sustained blood pressure reduction was observed in healthy human subjects. NO may provide the missing link, given its prominent role in vascular physiology, ever since it was recognized to be the vasodilatory substance EDRF. *In vitro* skin irradiation with UVA leads to NO release from dermal NO stores, which may act to lower blood pressure, although other mediators and processes could be involved. Supporting NO's role is the finding that systemic inhibition of NO formation leads to immediate increases in blood pressure. The potentially cardioprotective effect of NO production in response to UV light deserves further investigation; for the time being it should be interpreted warily due to concurrent skin cancer risk enhancement by prolonged time spent in direct sunlight [56].

## NO as a cosmeceutical

Aging and extensive exposure to environmental factors, including sun radiation, affect the skin in such a way that it undergoes structural and functional changes, which result in many of the characteristics of aged skin, including:

Loss of elasticity;
Wrinkle formation;
Loss of water-holding capacity;
Uneven fat distribution;
Cellulites;
Sagging.

Many of the above end points are the targets of cosmetic treatments. Through its regulation of melanogenesis and biostructural elements of the skin, NO could represent a potent cosmeceutical with legitimate biological activity conferring both beautifying and protective effects.

Reactive oxygen species (ROS) produced by UVR, detectable within 15 min of exposure, stimulate release of proinflammatory cytokines and growth factors that disrupt skin's structural integrity: AP-1 and NFκB increase production of matrix metalloproteinases that break down extracellular matrix collagen and elastin fibers, and TGF-β decreases production of collagen (Figure 6). Upregulation of AP-1 and NFκB is also implicated in photocarcinogenesis [57].

In keeping with its dualistic nature, NO-releasing materials could be employed as non-UV-based tanning agents [13,58] that simultaneously reduce UV-induced photodamage and skin cancer risk, owing to NO's ability to:

Stimulate melanogenesis → enhance endogenous pigmentation, which is associated with decreased risk of skin cancer [53,59];
Act as an antioxidant → attenuate the deleterious effects of ROS on skin aging and carcinogenesis [57,60];
Increase type I collagen synthesis in fibroblasts → decrease wrinkle development, as photodegradation of type I fibers is thought to play a role in this process [61].

This development would mark a major public health achievement, reducing the significant risk incurred by the nearly 30% of non-Hispanic white female high school students who reported indoor tanning during the previous 12 months, while maintaining the desired cosmetic benefits [62].

**Table 1.** **Antimicrobial activities of nitric oxide-releasing platforms.**

**Platform**	**Advantages**	**Limitations**	**Active against**
Acidified nitrite creams	Easily applied	Ingredients must be mixed immediately before application	*Burkholderia cepacia*
		Skin irritation	*Mycobacterium ulcerans*
			*Propionibacterium acnes*
			*Pseudomonas aeruginosa*
			*Staphylococcus aureus* (inc. MRSA)
			*Tinea pedis*
			*Trichophyton* spp.
Diazeniumdiolates (NONOates)	Easily produced	Risk of methemoglobin formation and release of toxic and carcinogenic byproducts	*Candida albicans* (DETA-NO)
	Stable under ambient conditions		*Escherichia coli* (β-Gal-NONOate)
	Spontaneous release of NO in predictable and dependable fashion		
gNO	Little *in vitro* toxicity to human skin cells	Expensive	*E. coli*
		Difficult to handle	*C. albicans*
		Requires gas cylinders for delivery	*P. aeruginosa*
		Extended duration of treatment requiring nonambulation	*S. aureus* (inc. MRSA)
		Cannot be exposed to oxygen	Group B Streptococcus
		Potential host toxicity via NO_2_ production and methemoglobinemia development	
NO-releasing nanoparticles (NO-np)	Ease of synthesis, storage, and administration		*Acinetobacter baumannii*
	Sustained NO retention and release		*C. albicans*
	Modifiable total NO quantity and rate of release		*Enterococcus faecalis*
	Minimal cutaneous and systemic toxicity		*E. coli*
			*Klebsiella pneumoniae*
			*P. aeruginosa*
			*S. aureus* (inc. MRSA)
			*Staphylococcus epidermidis*
			*Trichophyton mentagrophytes*
NO probiotic patch	Inexpensive	Patch-to-patch variability of gNO production depending on activity of *Lactobacillus fermentum* in each patch	*A. baumannii*
			*E. coli*
			*P. aeruginosa*
			*S. aureus* (inc. MRSA)
			*T. mentagrophytes*
			*Trichophyton rubrum*
Organic nitrates and nitrites (nitroglycerin, isosorbide mononitrate, sodium nitroprusside)	Long history of use	Limited knowledge of antimicrobial capabilities	Limited antibacterial properties
	Side effects well known	Tolerance development after prolonged use of nitrates	
		Cyanide production by sodium nitroprusside	
*S*-nitrosothiols (RSNOs)	Tissue selective	Require refrigeration in powder form prior to use	*Acanthamoeba castellanii*
	Can be designed to release NO at specified rates	Lack stability required for localized/topical delivery	*Enterobacter aerogenes*
			*E. coli*
			*Leishmania* spp.
			*Plasmodium falciparum*
			*P. aeruginosa*
			Coagulase-negative staphylococci
			*Salmonella typhimurium*
			*Serratia marcescens*
			*S. aureus*
			*Trypanosoma cruzi*
Zeolites (NO-metal complexes)	Stable	Lack of investigation into potential use in SSTI	*Bacillus subtilis*
	Modifiable NO release rate		*Clostridium difficile*
			*E. coli*
			*P. aeruginosa*
			*S. aureus* (inc. MRSA)

gNO: Gaseous nitric oxide; MRSA:Methicillin-resistant Staphylococcus aureus; NO: Nitric oxide; NONOate: Diazeniumdiolates; RSNO: Reactive nitrogen oxide species; SSTI: Skin and soft-tissue infection.

**Executive summary**

NO is produced by one of three nitric oxide synthase (NOS) isoforms present in nearly all types of skin cells and functions in establishing the skin's protective barrier, circulation and UV-stimulated melanogenesis and erythema.
Inappropriate inducible NOS (iNOS) upregulation is implicated in a multitude of dermatologic disease states, and NO deficiency may be involved in vasospastic conditions such as Raynaud phenomenon.
When stimulated by bacterial products and inflammatory cytokines, iNOS produces large amounts of NO to create reactive nitrogen oxide species (RNOS), which possess antimicrobial functions.
NO is being evaluated as a potential broad-spectrum topical antimicrobial agent with activity against Gram-positive and – negative bacteria, fungi and parasites.
To date, no resistance to NO's microbicidal activity has been demonstrated.
Hybrid nitric oxide nanoparticles (NO-np) exhibited strong bacterio-static and -cidal activity against many Gram-positive and -negative bacteria as well as fungi, both *in vitro* and *in vivo* in murine wound, burn and abscess models.
NO-np combined with glutathione (GSH) generate *S*-nitrosoglutathione (GSNO) – an NO-donating compound – and may represent an even more powerful antimicrobial agent.
NO's role in the skin's response to UV radiation and skin pigmentation may pave the way for future use as a cosmeceutical and/or novel non-UV-based tanning agent capable of reducing photoaging and skin cancer risk.
